# Use of anti-dementia drugs in home care and residential care and associations with neuropsychiatric symptoms: a cross-sectional study

**DOI:** 10.1186/s12877-015-0102-4

**Published:** 2015-08-13

**Authors:** Marja Kuronen, Hannu Koponen, Irma Nykänen, Pertti Karppi, Sirpa Hartikainen

**Affiliations:** Mikkeli Central Hospital, Porrassalmenkatu 35-37, FI-50100 Mikkeli, Finland; Department of Psychiatry, Institute of Clinical Medicine, University of Helsinki and Helsinki University Hospital, P. O. BOX 590, FI-00029 HUS Helsinki, Finland; School of Pharmacy, University of Eastern Finland, Kuopio, Finland; Faculty of Health Sciences, Kuopio Research Centre of Geriatric Care, P. O. BOX 1627, Kuopio, FI-70211 Finland

## Abstract

**Background:**

The number of people with dementia is increasing alongside the aging population, and most of these patients manifest with neuropsychiatric symptoms (NPS). The objective of this study was to investigate anti-dementia drug use and its associations with NPS.

**Methods:**

Questionnaires on demographic information, current drug use, activities of daily living and NPS were sent to all municipal home care producers and to all institutions providing long-term residential care in the South Savo Hospital District, Finland.

**Results:**

The study population comprised 2821 persons. Their mean age was 81 years and 68 % were female. Dementia had been diagnosed in 31 % (*n* = 410) in home care and in 56 % (*n* = 774) in residential care. Anti-dementia drugs were used by 69 % of patients with dementia. Hyperactivity symptoms were common in residential care patients (*n* = 456, 33 %), while problems with mood and apathy dominated in home care patients (*n* = 486, 54 %). In multivariate regression analysis, the mood symptoms and apathy subgroup was associated with use of an acetylcholinesterase inhibitor (AChEI) (OR 1.44; 95 % Cl 1.03–2.02), memantine (OR 1.77, 95 % Cl 1.15–2.72) or their combinations (OR 1.56, 95 % Cl 1.03-2.34). Hyperactivity symptoms were associated with combination therapy of this type (OR 2.03, 95 % Cl 1.36–2.34).

**Conclusions:**

The use of anti-dementia drugs was common in both care settings. The use of any anti-dementia drug or combination was associated with the mood and apathy subgroup. The hyperactivity subgroup was associated with combination use of memantine and AChEI.

## Background

Dementia contributes one-tenth of the years spent with disability in people aged over 60 years. This is more than the proportion of stroke, cardiovascular disease or cancer [1]. An estimated 42.3 million people globally will suffer from dementia by the year 2020 and over 80 million by 2040 [1], not only in high-income but also low- and middle-income countries [2]. The annual costs of dementia are estimated to be 600 billion US dollars [3]. Neuropsychiatric symptoms (NPS) occur in 80–90 % of persons with dementia [4]. They may be more disruptive to patients and their caregivers than the decline of cognition [5]. NPS are associated with decline in global functioning, increased use of medications and frequent hospitalization [6]. Agitation, aggression and psychosis are the most distressing NPS and correlate with early transfer to institutional care [7].

Many attempts have been made to form subgroups or symptom clusters of NPS [8–12]. In a large multicenter study with 2 354 outpatients with Alzheimer’s disease, four NPS subgroups were found: hyperactivity, psychosis, affective symptoms and apathy [13]. Petrovics and co-workers identified four factors based on the Neuropsychiatric Inventory (NPI) [14], namely psychosis factor, psychomotor factor, mood liability factor and instinctual factor [10]. Most studies agree that NPS form three to five subsyndromes consisting of hyperactivity symptoms, mood symptoms, psychotic symptoms and apathy [12, 13].

Anti-dementia drugs are used not only to improve cognitive functions but also to treat behavioral symptoms [15]. They are either AChEIs (donepezil, rivastigmine and galantamine) or memantine. AChEIs are recommended for use in mild to moderate dementia and to reduce NPS, and memantine in moderate to severe dementia and to diminish behavioral symptoms [16]. The concomitant use of memantine and AChEI is not recommended by NICE 2011, although there may be a small benefit on NPS at six months after the initiation of treatment [17]. Most studies exploring the effect of anti-dementia drugs on NPS have been primarily designed to evaluate their effect on cognition [18]. The effect on NPS may be limited [19].

As the number of people with dementia is increasing and most of these patients manifest with NPS, the aim of this study was to investigate the use of anti-dementia drugs and the prevalence of NPS in two different populations and the associations between anti-dementia drug use and NPS.

## Methods

### Study design and participants

**Fig. 1:**
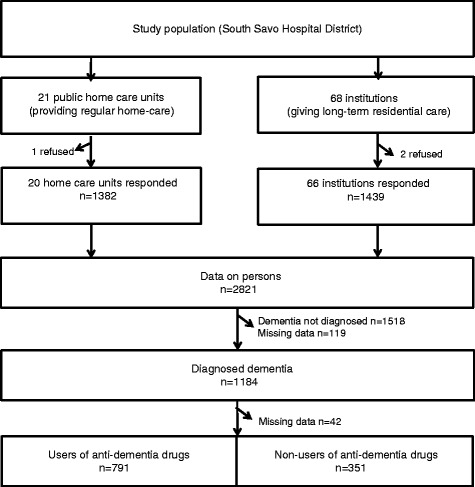
Flowchart of the study

We identified with the help of local and regional authorities all public home care units (*n* = 21) providing regular care (a nurse visiting a home-dwelling patient at least once a week) and all institutions (*n* = 68) giving long-term residential care to older people, including both private and municipal residential care facilities, nursing homes and long-term wards in municipal hospitals. The catchment area of the South Savo Hospital District is 105 000 inhabitants [20]. Due to strong support from the local authorities, we had an excellent response rate. Twenty out of 21 municipal home care units responded, and 66 out of 68 residential care units responded (Fig. 1).

General information about the study and the questionnaires were mailed to the nurses and doctors in charge who were responsible for instructing the nurses on the field. Written instructions on how to carry out the assessments were included, and the staff were given the name and telephone number of the first writer if additional guidance was needed. The study was carried out in May 2011.

### Questionnaires

The basic demographic information of each patient, e.g. municipality, service unit or residential care unit, age and sex, was followed by questions concerning current regularly used medications, which were obtained from electronic medical records. Activities of daily living (ADL) were assessed by the Barthel Index (scale 0–100) [21]. On this scale, the higher the score, the better the functioning. Cognitive functioning was assessed in two ways. Firstly, we inquired about the results of the latest Mini Mental State Examination (MMSE, scale 0–30) [22] if carried out in 2010 or 2011. Cognition had been assessed by MMSE in 743 patients (51.6 %) in residential care and in 627 patients (45.2 %) in home care services. Nurses reported whether dementia had been diagnosed by a physician, but we did not sort out different dementia types. Secondly, we asked the nurse to assess each patient’s memory by direct observation and assign it to one of four categories by the clinical dementia rating (normal, slightly impaired, moderately impaired, or severely impaired) [23]. We did not ask the dates of dementia diagnosis, start of the anti-dementia drugs or the beginning of NPS.

Each patient was evaluated by a nurse who knew the patient, and NPS were listed according to a symptom list based on the Neuropsychiatric Inventory (NPI). The scale was originally developed to assess behavioral and psychological symptoms in dementia and it consists of 12 items, each of which is scored for frequency and severity. Nurses reported all NPS of each patient during the preceding week in home care (due to visiting procedures at least once a week in home care) and during the preceding 24 h in residential care (observation and care available 24/7). The assessment gave only the presence or absence of the symptom, not the severity or the effects on carers. The interrater reliability was not assessed, but the nurses had written instructions to evaluate the symptoms. We organized the NPS into three subgroups [8]: 1) hyperactivity consisting of agitation or aggression, disinhibition, irritability and aberrant motor behaviour, 2) psychosis consisting of delusions and hallucinations, and 3) mood symptoms and apathy consisting of depression, anxiety, sleeping disturbances, eating disturbances, apathy and euphoria. Fifty-three patients in home care and 89 patients in residential care had no data concerning NPS. These 142 patients were excluded from all NPS or subgroup analyses.

### Classification of medication

Drugs were classified according to the Anatomical Therapeutic Chemical (ATC) classification of medicines recommended by the World Health Organization (WHO) [24]. The anti-dementia drugs included memantine (N06DX01) and the AchEIs: (N06D) donepezil (N06DA02), rivastigmine (N06DA03) and galantamine (N06DA04).

### Ethics

The study design was accepted by the Ethics Committee of the South Savo Hospital District. The use of current medication was obtained from medical records, without identification of individual patients. Other data were obtained from a special questionnaire form, which was sent to the institutions and home care services. Informed consents were not needed in our study, because data did not contain birth dates or other material by which a person could have been identified. In addition, ad hoc questionnaire did not include other identification than municipality, service unit or residential care unit, age and gender. As participation in the study was supported by local health authorities, no incentives were needed to promote the response rate.

### Data analysis

Differences in characteristics of patients by setting, prevalence and number of NPS among users of anti-dementia drugs were described using proportions and means with standard deviation (SD). Statistical comparisons between groups were conducted using Chi-square test and independent samples *t*-test or one-way analysis of variance, with p ≤ 0.05 considered significant. Univariate and multivariate (stepwise, forward selection) regression analyses were performed to identify demographic (age, sex and home care/residential care) and neuropsychiatric subgroups (hyperactivity, psychotic symptoms, mood symptoms and apathy) associated with anti-dementia drug use. Results were expressed as odds ratios (ORs) with corresponding 95 % confidence intervals (95 % CIs). In the Tables 3 and 4 the missing *p*-values of NP were due to confidentiality matters. Data were analyzed using SPSS 19.0 software.

## Results

The study population comprised 2821 persons, 68.1 % (*n* = 1921) of whom were women (Table 1). The mean age of participants was 80.9 (SD 10.1) years, 82.0 (SD 9.8) years in residential care and 79.8 (SD 10.4) years in home care. Only 8 % (*n* = 224) were younger than 65 years. Of home care patients, 81 % lived alone. Dementia had been physician-diagnosed in 1184 patients (43.8 %), 56.0 % in residential care and 31.1 % in home care. ADL were worse in residential care (mean Barthel Index 36.9, SD 30.8) than in home care (mean 80.8, SD 20.9, *p* < 0.001). Bedridden persons constituted 0.9 % of patients in home care, and 15.4 % in residential care. Anti-dementia drugs were used by 31.9 % (*n* = 901) of the study population. AChEIs were used by 19.3 % of patients and memantine by 8.7 % of residential care and 3 % of home care patients. Combination therapy with AChEI and memantine was used by 9.0 % of residential care and 4.4 % of home care patients.

**Table 1:** Characteristics, functioning and anti-dementia drug use of patients by setting

	Total	Residential care	Home care	*P*- value
	(*n* = 2821)	(*n* = 1439)	(*n* = 1382)	
Characteristics				
Female, n (%)	1921 (68.1)	995 (69.1)	926 (67.0)	0.223
Mean age, years (SD)	80.9 (10.1)	82.0 (9.8)	79.8 (10.4)	<0.001
≤ 64, n (%)	224 (8.0)	89 (6.2)	135 (10.6)	
65-74, n (%)	354 (12.6)	172 (11.0)	182 (12.5)	
75-84, n (%)	1056 (37.6)	511 (35.6)	545 (39.7)	
≥ 85, n (%)	1174 (41.8)	664 (46.2)	513 (37.2)	
Diagnosis and functioning				
Diagnosed dementia, n (%)	1184 (43.8)	774 (56.0)	410 (31.1)	<0.001
ADL score (%, SD)	58.4 (34.3)	36.9 (30.8)	80.8 (20.9)	<0.001
Bedridden, n (%)	233 (8.3)	221 (15.4)	12 (0.9)	<0.001
Drug use				
Total number of drugs, mean (SD)	8.6 (4.7)	8.5 (4.2)	8.5 (5.3)	0.437
Anti-dementia drug users, n (%)	901 (31.9)	516 (35.9)	385 (27.9)	0.007
AChEI alone, n (%)	545 (19.3)	262 (18.2)	283 (20.5)	0.015
Donepezil, n (%)	291 (10.3)	127 (8.8)	161 (11.6)	
Rivastigmine, n (%)	153 (5.4)	99 (6.9)	54 (3.9)	
Galantamine, n (%)	107 (3.8)	36 (2.5)	69 (4.9)	
Memantine alone, n (%)	166 (5.8)	125 (8.7)	41 (3.0)	0.003
AChEI and memantine	190 (6.7)	129 (9.0)	61 (4.4)	<0.001
Donepezil + memantine, n (%)	94 (3.3)	60 (4.2)	34 (2.5)	
Rivastigmine + memantine, n (%)	63 (2.2)	51 (3.5)	12 (0.9)	
Galantamine + memantine, n (%)	33 (1.2)	18 (1.3)	15 (1.1)	

Chi-square test for categorical variables and Student’s *t*-test for continuous variables

ADL score = Barthel Index, scale 0–100

*AChEI* Acetylcholinesterase inhibitor

### Neuropsychiatric symptoms

More than half of the patients in both settings suffered from NPS (Table 2). In residential care 19.1 % and in home care 15.1 % of patients suffered from two to three simultaneous NPS, and 5.7 % and 4.5 % suffered from at least four symptoms, respectively. The most common NPS subgroup was mood symptoms and apathy (*n* = 908). Hyperactivity subgroup symptoms occurred in 684 patients and psychotic symptoms in 278 patients. In residential care, the most common subgroup was hyperactivity (32.9 %), while in home care mood symptoms and apathy predominated (54.4 %). The most common distinct symptoms were agitation/aggression, irritability and depression in residential care, but depression, sleeping problems and irritability in home care. Disinhibition was seldom seen (2.0 %) in home care, whereas it was reported in almost one-tenth (8.9 %) of patients in residential care.

**Table 2:** Frequency of NPS (n, %) and subgroups in patients by setting

	Residential care	Home care	*P*-value
	*n* = 1386 (%)	*n* = 1293 (%)	
Frequency of NPS			
None	643 (46.4)	642 (49.7)	0.014
One symptom	398 (28.7)	397 (30.7)	0.357
Two to three symptoms	265 (19.1)	195 (15.1)	0.002
Four or more symptoms	79 (5.7)	58 (4.5)	0.110
Subgroups			
Hyperactivity^a^	456 (32.9)	228 (17.6)	<0.001
Agitation/aggression	224 (16.1)	92 (7.1)	
Disinhibition	123 (8.9)	26 (2.0)	
Irritability	220 (15.9)	144 (11.1)	
Aberrant motor behaviour	83 (6.0)	21 (1.6)	
Psychosis^a^	146 (10.6)	132 (10.2)	0.788
Delusions	72 (5.2)	76 (5.9)	
Hallucinations	93 (6.7)	77 (6.0)	
Mood symptoms and apathy^a^	422 (30.5)	486 (54.4)	<0.001
Depression	157 (11.3)	271 (21.0)	
Anxiety	115 (8.3)	101 (7.8)	
Sleeping problems	130 (9.4)	179 (13.8)	
Eating problems	75 (5.4)	70 (5.4)	
Elation	20 (1.4)	18 (1.4)	
Apathy	69 (5.0)	40 (3.1)	

*NPS* Neuropsychiatric symptoms

^a^One person could suffer from several symptoms, thus total number of cases in the subgroups is not the same as the total number of patients suffering from at least one neuropsychiatric symptom (NPS). Missing cases: 53 in residential care and 89 in home care

### Patients with diagnosed dementia

Of persons with diagnosed dementia (*n* = 1142), altogether 66.8 % (*n* = 791) used anti-dementia drugs (Table 3). Of these 58.8 % used AChEIs, 18.3 % memantine and 22.9 % combinations of an AChEI and memantine. Users of anti-dementia drugs had higher mean ADL (58.0 vs. 29.8) and MMSE score (16.0 vs.14.7) than non-users. The prevalence of NPS was about the same in both groups.

**Table 3:** Frequency of NPS and subgroups, ADL functioning and cognition among patients with diagnosed dementia

	Users of anti-dementia drugs	Non-users of anti-dementia drugs	*P*-value*
	*n* = 791	*n* = 351	
Frequency of NPS, n (%)			
None	320 (40.4)	162 (46.1)	0.074
One symptom	252 (31.8)	114 (32.5)	0.867
Two to three symptoms	171 (21.6)	55 (15.7)	0.016
Four or more symptoms	48 (6.1)	20 (5.7)	0.771
Any NPS, n (%)	471 (59.5)	189 (53.8)	0.771
Subgroups, n (%)			
Hyperactivity	264 (33.3)	116 (33.0)	
Psychosis	105 (13.2)	36 (10.3)	
Mood symptoms and apathy	299 (37.6)	90 (25.6)	
ADL score, mean (SD)	58.0 (31.5)	29.8 (34.5)	<0.001
MMSE, mean (SD)	16.0 (6.6)	14.7 (8.0)	0.011

ADL = Barthel Index, scale 0–100

*NPS* Neuropsychiatric symptoms, *MMSE* Mini Mental State Examination

*Chi-square test for categorical variables and Student’s *t*-test for continuous variables

The frequency of NPS did not differ between the three groups studied: users of AChEI only, memantine only or combination of both (Table 4). In multivariate analyses, any kind of anti-dementia drug use appeared to be independently associated with the subgroup of mood symptoms and apathy, and the use of a combination therapy was associated with hyperactivity but not with psychotic symptoms (Table 5). The ADL score was associated with any kind of anti-dementia drug use, and age was associated with use of either an AChEI or memantine, but not with their combination.

**Table 4:** Frequency of NPS and subgroups, ADL functioning and cognition by use of anti-dementia drugs

	AChEI only	Memantine only	AChEI and memantine	*P*-value*
	*n* = 465	*n* = 145	*n* = 181	
Frequency of NPS, n (%)				
None	202 (43.4)	60 (41.4)	58 (32.0)	0.065
One symptom	144 (31.0)	47 (32.4)	61 (33.7)	0.569
Two to three symptoms	97 (20.9)	28 (19.3)	46 (25.4)	0.296
Four or more symptoms	22 (4.7)	10 (6.9)	16 (8.8)	0.107
Any NPD, n (%)	263 (56.6)	85 (58.6)	123 (68.0)	0.029
Subgroups, n (%)				
Hyperactivity	136 (29.2)	45 (31.0)	83 (45.9)	
Psychosis	54 (11.6)	18 (12.4)	33 (18.2)	
Mood symptoms and apathy	170 (36.6)	57 (39.3)	72 (39.8)	
ADL score, mean (SD)	62.3 (31.5)	48.0 (31.7)	54.6 (28.9)	<0.001
MMSE, mean (SD)	17.2 (6.3)	14.4 (7.2)	13.9 (6.2)	<0.001

ADL = Barthel Index, scale 0–100

*NPS* Neuropsychiatric symptoms, *AChEI* Acetylcholinesterase inhibitor, *MMSE* Mini Mental State Examination

*Chi-square test for categorical variables and one-way analysis of variance for continuous variables

**Table 5:** Univariate and multivariate associations between patient characteristics, subgroups and anti-dementia drug use

Variable	AChEI only		Memantine only		AChEI + memantine	
	Univariate	Multivariate^a^	Univariate	Multivariate^a^	Univariate	Multivariate^a^
	OR (95 % CI)	OR (95 % CI)	OR (95 % CI)	OR (95 % CI)	OR (95 % CI)	OR (95 % CI)
Age (years)	1.02 (1.00 to 1.04)	1.04 (1.01 to 1.06)	1.03 (1.00 to 1.07)	1.05 (1.02 to 1.08)	0.99 (0.96 to 1.01)	
Female sex	1.06 (0.78 to 1.43)		1.48 (0.93 to 2.33)		1.00 (0.67 to 1.48)	
ADL score	1.03 (1.02 to 1.03)	1.03 (1.02 to 1.04)	1.01 (1.01 to 1.02)	1.02 (1.01 to 1.03)	1.02 (1.01 to 1.03)	1.02 (1.02 to 1.04)
Residential care	0.31 (0.24 to 0.40)		1.37 (1.04 to 1.80)		1.13 (0.79 to 1.56)	
NPS subgroups						
Hyperactivity	0.83 (0.61 to 1.12)		0.91 (0.60 to 1.38)		1.69 (1.17 to 2.45)	2.03 (1.36 to 3.04)
Psychosis	1.15 (0.73 to 1.97)		1.24 (0.67 to 2.26)		1.93 (1.16 to 3.23)	
Mood symptoms and apathy	1.66 (1.22 to 2.25)	1.44 (1.03 to 2.02)	1.87 (1.24 to 2.83)	1.77 (1.15 to 2.72)	1.91 (1.30 to 2.80)	1.56 (1.03 to 2.34)

ADL score = Barthel Index, scale 0–100

*AChEI* Acetylcholinesterase inhibitor, *NPS* Neuropsychiatric symptoms, *OR* odds ratio, *CI* confidence interval

^a^Forward selection. Variables included in the multivariate model are shown

## Discussion

In our large, geographically defined study population, the use of any anti-dementia drug was associated with the NPS subgroup of mood symptoms and apathy. In addition, hyperactivity subgroup symptoms were associated with the combination therapy of AChEI and memantine. Psychotic symptoms were not associated with the use of AChEIs, memantine or their combinations. To our knowledge, this is the only study investigating the associations between anti-dementia drug use and prevalence of NPS subgroups. Mood symptoms and apathy were the most common NPS in anti-dementia drug users. According to previous studies, anti-dementia drugs improve depressive symptoms in mild to moderate dementia, independent of any effect on cognition [25]. A Swedish study [26] reported no association between depressive symptoms and the use of anti-dementia drugs. Instead, they found an association between anti-dementia drug use and aggressive behaviour. The efficacy of AChEIs in the management of NPS in Alzheimer’s disease is limited [14]. Despite, anti-dementia drugs are recommended as a first-line pharmacological treatment for NPS just after non-pharmacological interventions [20]. In Finland, the National Treatment Guidelines recommend the use of AChEIs to treat NPS in patients with dementia [16].

Combination therapy of memantine and AChEI has growing evidence of efficacy in cognitive decline [27, 28], but the effect on NPS is controversial [29]. However, memantine alone has had some effect on hyperactivity symptoms [7]. Psychotic symptoms occurred in every tenth patient in our study. Although memantine has been found to have some benefits in treating psychotic symptoms [30] without an increased risk of mortality, which occurs with anti-psychotics [31], we found no association between the use of any anti-dementia medication and the psychosis subgroup. Persons with psychotic symptoms may have been treated with anti-psychotic drugs rather than with anti-dementia drugs. This may be due to evidence that risperidone, olanzapine and aripiprazole are superior to placebo in treating agitation and psychosis in dementia [32].

The use of anti-dementia drugs has increased in the last few years [33], and the proportion of AChEI users in long-term residential care varies from 3 % in Australia to 30 % in the United States [34, 35]. In Finland, during 2005–2009, 84 % of patients with Alzheimer’s disease used AChEI and 47 % memantine, 22 % using both of these concomitantly [36]. In our study, donepezil was the most commonly used anti-dementia drug, consistent with previous studies [36]. The use of memantine or combination therapy was common also in our study, whereas in Sweden, memantine was used in 2007 by only 3.1 % of patients with cognitive impairment [26].

The main strength of our study is the nearly 100 % coverage of the targeted study population, receiving long-term residential care or regular home care services in the South Savo Hospital District. We obtained comprehensive data concerning drug use, NPS profile and basic demographic characteristics of these patients, enabling a representative overview of the use of anti-dementia drugs and NPS in two different care settings. The study has also some limitations. We did not ask the dates of dementia diagnosis, start of the anti-dementia drugs or the beginning of NPS. This cross-sectional design forms a limitation, and allows us only to evaluate the correlations between NPS and use of anti-dementia drugs at a spesific time point. The questionnaires also gave only an approximation of NPS, as the interrater reliability was not assessed, and nurses had only written instructions for evaluating the symptoms of patients. The assessment of only the presence or absence of the symptom forms a limitation. Different time windows to detect NPS in home care and residential care (previous week vs. preceding 24 h) due to different observational possibilities may also have affected the prevalence of NPS to some extent. However, there are only a few studies about NPS in home-dwelling persons with dementia [37] and there are, to our knowledge, no other studies detecting NPS in a almost all persons receiving home care in a defined area.

## Conclusions

The use of anti-dementia drugs was common both in long-term residential care and home care. The use of AChEI and/or memantine was associated with the mood and apathy subgroup and combination therapy with the hyperactivity subgroup symptoms. As clinicians, we need more studies on the effectiveness of anti-dementia drugs to ensure effective and safe pharmacotherapy for our vulnerable patients with dementia.

## Abbreviations

AChEI: Acetylcholiesterase inhibitor
ADL: Activities of daily living
ATC: Anatomical therapeutic chemical
CI: Confidence interval
MMSE: Mini mental state examination
NMDA: N-methyl-D-aspartate
NPI: Neuropsychiatric Inventory
NPS: Neuropsychiatric symptoms
OR: Odds ratio
SD: Standard deviation
WHO: World Health Organization
